# Trust in Science, Perceived Vulnerability to Disease, and Adherence to Pharmacological and Non-pharmacological COVID-19 Recommendations

**DOI:** 10.3389/fpsyg.2021.664554

**Published:** 2021-04-30

**Authors:** Ivana Hromatko, Mirjana Tonković, Andrea Vranic

**Affiliations:** Department of Psychology, Faculty of Humanities and Social Sciences, University of Zagreb, Zagreb, Croatia

**Keywords:** protection motivation theory, adherence to protective measures, behavioral immune system, perceived vulnerability, trust in science

## Abstract

Protection motivation theory (PMT) is a theoretical framework informative for understanding behavioral intentions and choices during exceptional and uncommon circumstances, such as a pandemic of respiratory infectious disease. PMT postulates both the threat appraisal and the coping appraisal as predictors of health behaviors. Recent advances in the field of behavioral immune system (BIS) research suggest that humans are equipped with a set of psychological adaptations enabling them to detect the disease-threat and activate behavioral avoidance of pathogens. The present study, set within PMT framework and informed by the BIS research, aimed to explain and predict voluntary adherence to COVID-19 guidelines by perceived personal risk and vulnerability to disease as threat appraisal variables, and trust in science as the response efficacy element of coping appraisal. Gender, age, belief in the second wave, perceived personal risk, germ aversion, and trust in science were all found to be significant positive predictors of the intent to adhere to non-pharmacological COVID-19 recommendations, with the belief in the second wave, germ aversion, and trust in science being the most important ones. On the other hand, only the belief in the second wave and trust in science were significant positive predictors of the intent to adhere to pharmacological COVID-19 recommendations (i.e., to vaccinate). Interventions aimed at enhancing preventative measures adherence should take into account that the psychological mechanisms underlying adherence to these two types of recommendations are not identical.

## Introduction

While scientific and pharmacological efforts are globally being put forth into the development and distribution of vaccines, non-pharmacological recommendations (NPR, e.g., handwashing, physical distancing) remain a key individual mean of limiting COVID-19 (World Health Organization, 2020a). As documented in previous pandemics, public consent to WHO guidelines is challenging to achieve (Gilles et al., 2011; Shultz et al., 2015). According to health behavior theories, the adherence to NPR is determined by the cost-benefit analysis of recommended behaviors. Individual determinants of preventive behavior span from demographics to attitudes and beliefs (e.g., Clark et al., 2020). Given the high infectivity of COVID-19 (leading to 122 M cases and 2.69 M deaths worldwide, as of March 19th; WHO), the potential harm caused by even a single non-compliance with NPR can have exponential negative effects for a number of persons. Even with an unlikely scenario of world-wide high adherence to NPR, further spread of pandemic cannot be seized without a satisfactory vaccination rate (estimated at 70% of the population; Randolph and Barreiro, 2020). Given the surge in global anti-vaccination and anti-scientific movements (Hussain et al., 2018; Hotez, 2020), this endeavor proves to be even more difficult.

When a global plan is founded on individual actions, promoting the understanding of individual adherence to health guidelines is of utmost importance – it could improve informing the public and in turn raise adherence. Also, such insights are instrumental for future health prevention programs. In the present study, we have examined individual demographics and beliefs regarding COVID-19, set within the protection motivation theory (PMT, e.g., Floyd et al., 2000; Boer and Mashamba, 2005; Al-Rasheed, 2020; Margraf et al., 2020) and informed by the behavioral immune system (BIS) research (Schaller, 2016). PMT framework is crucial in understanding behavioral choices during exceptional and uncommon circumstances, with epidemics of respiratory infectious disease being among the most serious of them (e.g., Williams et al., 2015). In so far, PMT has served to explore preventive behaviors related to the seasonal influenza vaccination (Ling et al., 2019), sun-safe behavior (Lowe et al., 2000; Moeini et al., 2019), and SARS preventive behaviors (Jiang et al., 2009). According to PMT, protective motivation depends on the threat and coping appraisal.

*Coping appraisal* taps beliefs about risk minimization, either at individual (such as perceived self-efficacy or one’s own perceived coping resources; e.g., Milne et al., 2000) or group level response (such as trust in policy-makers or science; e.g., Plohl and Musil, 2020). Response efficacy concerns beliefs that adopting a particular behavioral response will be effective in reducing the diseases’ threat (Van der velde and Van der Plight, 1991) and is operationalized by linking consequences and their likelihood to the recommended behavior (Lwin and Saw, 2007). Given the growing world-wide anti-scientific sentiment – perils of which became obvious during the COVID-19 pandemic (e.g., distrust in scientific authorities regarding the face-masks, social distancing, asymptomatic transmission, and above all vaccine’s safety and efficacy), we opted to explore the relation between (mis)trust in both, science as an epistemic process and scientists as those conducting it, and the self-reported intent to adhere to the official COVID-19 guidelines. As previous studies suggest (e.g., Organization for Economic Co-operation and Development, 2020; Plohl and Musil, 2020), individuals placing trust in expert decision-makers will be more likely adhere to the guidelines. In the context of the coping appraisal within the PMT, we hypothesized that individuals with higher scores on the *Trust in Science and Scientists Inventory* (TSSI; Nadelson et al., 2014) will follow the COVID-19 guidelines more diligently.

*Threat appraisal* regards personal beliefs about the likelihood of contracting a disease and/or perceived vulnerability or risk. We opted to explore this part of the PMT in relation to the BIS. Stemming from the evolutionary psychology framework, BIS is defined as a set of cognitive and affective mechanisms (psychological adaptations) which enable detection of potential pathogens in the immediate environment and trigger avoidant and prophylactic behaviors (Schaller, 2006, 2016). BIS has been extensively studied on the perceptual (detection of pathogens ranging from perceived sources of contamination in public toilets to detection of subtler cues of illness among conspecifics) or affective-cognitive level (emotions and cognitions related to BIS-activation; i.e., negative emotions and avoidance motivations). In this study, we investigated the behavioral correlate of BIS activation (i.e., adherence to the COVID-19 guidelines). BIS activation is largely emotion-driven thus often unconscious and automatic. Yet, triggered prophylactic behaviors also include rational, conscious choices, such as vaccination or avoidance of public transportation during the flu season (Schaller, 2016).

The general purpose of BIS is the avoidance of pathogens and infective carriers and the expression of such adaptation is expected throughout the whole species. However, individuals vary regarding the BIS reactivity, and studies suggest these variations are related to one’s health status. For example, recently and frequently ill people show greater BIS activation (Stevenson et al., 2009; Miller and Maner, 2011; Murray et al., 2019), as do pregnant women during the first trimester (Navarrete et al., 2007), and individuals with gene variants associated with greater susceptibility to certain infectious diseases and poorer immunological function (MacMurray et al., 2014; Napolioni et al., 2014).

While perceived infectability refers to one’s own susceptibility to infection, germ aversion covers behaviors exerting emotional discomfort in high pathogen context, in turn deterring from the source of infection. These two pathogen avoidance tendencies jointly measure perceived vulnerability to disease (Duncan et al., 2009) and are often operationalized as a trait. However, group level scores on disgust sensitivity, germ aversion, and perceived infectability have risen significantly during the current unprecedented global health crisis (Hromatko et al., 2021; Miłkowska et al., 2021; Stevenson et al., 2021), indicating that heightened awareness of potential contamination cues might lead to a sensitization to pathogen threat, i.e., greater (re)activity of BIS. The perceived vulnerability to disease is associated with stronger reactions to the COVID-19 threat, including increased anxiety, need for behavioral change, and higher importance of proactive behavior and social distancing (Makhanova and Shepherd, 2020). Converging onto the context of threat appraisal within the PMT, we hypothesized that individuals with higher scores on BIS-related variables (germ aversion and perceived infectability) and perceived personal risk (a one-item measure exploring whether participants perceived themselves to be at higher risk of COVID-19) will, again, be more diligent in following guidelines.

Finally, demographic variables could also affect adherence, either directly or indirectly *via* other important variables. Higher education and SES are predictive of trust in science (Nadelson et al., 2014; Peterlin, 2019). Women are more likely to engage in NPR and related health-behavior (Yıldırım and Güler, 2020; Yıldırım et al., 2021). Furthermore, women consistently score higher on disgust sensitivity (Al-Shawaf et al., 2018), which is central to BIS activation. However, age was not found related to voluntary adherence to NPR (Clark et al., 2020), although it was implicated in some BIS-related outcomes: for example, older participants preferred larger interpersonal distance during pandemic (Hromatko et al., 2021).

### Aim

**Figure 1:**
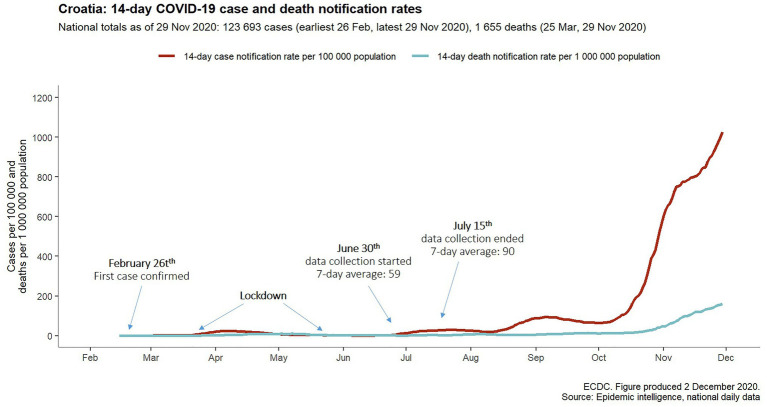
The data collection took part after successful mitigation of the first wave, and was preceded by 2 months of only sporadic new cases. (Source: COVID-19 Data Repository by the CSSE at Johns Hopkins University). (Source: ECDC).

We aimed to explain and predict voluntary adherence to COVID-19 guidelines *via* perceived personal risk and perceived vulnerability to disease as threat appraisal variables, and trust in science as the efficacy element of the coping appraisal. Since this study was conducted between the two waves of COVID-19 pandemic in Croatia, the adherence was operationalized as participants’ *intent* to adhere to recommendations *if/when the second wave occurs*. The rationale was led by the fact that the first wave was successfully mitigated with the most restrictive set of measures (acc. to the Oxford stringency index: Hale et al., 2020), and the data collection period was preceded by almost 2 months of sporadic new cases. Even though health authorities kept issuing warnings of high probability of the second wave, general public was only moderately convinced (see Figure 1). Therefore, we measured and controlled for belief in the possibility of the second wave, with a prediction that this variable will explain significant proportion of the variance in the intent to adhere to the guidelines. In all, we hypothesized that the higher both of the two types of appraisals (threat and coping), the higher the compliance with the COVID-19 guidelines.

## Materials and Methods

### Participants

Among 859 participants (375 male, 457 female, and 27 did not report gender), aged 16–73 (*M* = 28.18, *SD* = 11.81), 54.7% have finished elementary or high school, 21.1% had a bachelor degree, 17.8% had a graduate level degree (MA), and 6.3% a postgraduate degree (PhD).

### Procedure

The link to the questionnaire was posted on various social networks during first 2 weeks of June, 2020. As explained, this period was preceded by almost 2 months of only sporadic new cases, i.e., the number of newly confirmed 3-day moving average cases ranged from 0 to 3 throughout May and June (Worldometer, 2021), and took place at the mere onset of the second wave (Figure 1).

Upon given an informed consent, participants have proceeded to the initial questionnaire consisting of: (1) sociodemographic information, (2) a question about belief in the second wave and, (3) a question regarding the perceived personal risk. Following this, participants filled in the instruments described below.

### Instruments

#### Sociodemographic Information

Participants reported their gender, age, education (elementary school/high school/undergraduate level/master/postgraduate level), and the population of their place of residence (eight-point scale, ranging from below 2,000 to over 200,000 inhabitants).

*Belief in the second wave* was measured on a five-point scale grading one’s agreement with the statement *I believe that the second wave of the pandemic will come (or has already started)*.

#### Perceived Personal Risk

One-item in which participants were asked whether they or their significant others belong to the COVID-19 high-risk group (e.g., whether they are older or immunocompromised, have chronic disease, asthma or similar; importantly, we did not define a high-risk group; rather we allowed for participants’ self-assessment of whether they are likely to develop a complicated presentation of COVID-19 infection).

*Intent to Adhere to COVID-19 Preventive Measures Scale (IA-COVID-19)*^1^ is a 12-item scale developed for the purpose of this study to examine the adherence intent during the next wave of pandemic (for example: *If the second wave occurs, I will… avoid closed and crowded spaces*). Participants report their intent to adhere to each of measures on a five-point scale (1 = *I do not plan to do that*, 5 = *Yes, I will definitely do that*). Final analysis yielded one-factor solution with 10 items (*α* = 0.88), while two items with low loadings were omitted. One of these items was “*I will take a COVID-19 vaccine*,” which is an important health-related behavior during the pandemic, and we have investigated it separately thus differentiating between the intent to adhere to pharmacological vs. NPR.

#### Perceived Vulnerability to Disease Scale

The scale consists of two subscales: (1) *Perceived Infectability* (seven items), assessing beliefs about one’s susceptibility to infectious diseases and (2) *Germ Aversion* (eight items), assessing emotional discomfort in contexts that connote especially high potential for pathogen transmission. Participants indicate their agreement with the items on a seven-point scale (1 = strongly disagree, 7 = strongly agree) with mean result as a subscale score. Reliabilities of *Perceived Infectability Subscale* and *Germ Aversion Subscale* are *α* = 0.87 and *α* = 0.74, respectively (Duncan et al., 2009).

#### Trust in Science and Scientists Inventory

A 21-item scale was shortened to a 13-item form based on the previous studies (Peterlin, 2019). Responses are given on a five-point Likert scale (1 = extremely disagree; 5 = extremely agree) and such scale showed high internal validity (*α =* 0.88; Nadelson et al., 2014).

## Results

Descriptive statistics and correlations are shown in Table 1. Approximately, one half of participants (55.2%) considered themselves or their significant others to be in a COVID-19 high-risk group.

**Table 1:** Correlation matrix and descriptive statistics.

		*M*	*SD*	1	2	3	4	5	6	7	8	9	10
1	NPR adherence intent	3.65	0.87	—									
2	Intent to vaccinate	3.25	1.52	0.38^**^	—								
3	Gender			0.12^**^	−0.04	—							
4	Age	28.2	11.80	0.12^**^	−0.02	−0.08^*^	—						
5	Education	2.74	0.99	0.09^**^	0.03	−0.09^**^	0.63^**^	—					
6	Residence size	6.44	2.25	0.06	0.05	−0.13^**^	0.10^**^	0.18^**^	—				
7	Belief in the second wave	3.86	1.16	0.30^**^	0.24^**^	−0.02	−0.16^**^	−0.06	0.11^**^				
8	Perceived personal risk			0.15^**^	0.06	0.08^*^	−0.02	−0.01	<0.01	0.14^**^	—		
9	Perceived infectability	2.98	0.85	0.09^*^	−0.01	0.04	−0.03	−0.03	0.06	0.05	0.10^**^	—	
10	Germ aversion	4.27	0.93	0.27^**^	0.03	0.12^**^	0.10^**^	<0.01	−0.05	−0.02	0.01	0.19^**^	—
11	Trust in science and scientists	3.78	0.71	0.19^**^	0.37^**^	−0.10^**^	<−0.01	0.03	0.16^**^	0.21^**^	0.04	−0.05	−0.04

* *p* < 0.05;

** *p* < 0.01.

Originally, one hierarchical regression analysis was planned, with sociodemographics (gender, age, and education) entered as the first, threat appraisal variables [perceived vulnerability to disease scale (PVD) and perceived personal risk] as the second, and coping appraisal variables (measures efficacy, operationalized *via* TSSI) as the third block of predictors, with the intent to adhere as a criterion variable. However, as described in the instruments section, the IA-COVID-19 showed a two-factor structure, with majority of items loading onto the factor best described as adherence to NPR and the item about the intent to vaccinate loading onto a second factor; such a finding is easily interpretable as the vaccination is not a NPR. Therefore, we have conducted two regression analyses as described above; first with the intent to adhere to NPR as the criterion and the second with the intent to vaccinate as the criterion.

### Intent to Adhere to the NPR

As previously stated, we hypothesized that sociodemographics, belief in the second wave, perceived personal risk, perceived infectability, germ aversion, and trust in science and scientists can significantly contribute to individual differences in adherence to the COVID-19 recommendations. Sensitivity analysis conducted in G*Power 3.1 (Faul et al., 2009) revealed that with our sample of *N* = 859 participants, regression analysis with nine predictors has 0.9 power (*α* = 0.05) to detect small effect size of *f^2^* = 0.02 (Cohen, 1988). A three-step multiple regression analysis described above was conducted. Regression statistics are shown in Table 2.

**Table 2:** Summary of hierarchical regression analysis for variables predicting the intent to adhere to COVID-19 preventive measures.

	Step 1			Step 2			Step 3		
Variable	*B*	*SE*	*β*	*B*	*SE*	*β*	*B*	*SE*	*β*
Gender	0.26	0.06	15^**^	0.17	0.06	0.10^**^	0.20	0.06	0.11^**^
Age	0.01	<0.01	0.18^**^	0.01	<0.01	0.13^**^	0.01	<0.01	0.13^**^
Education	<0.01	0.04	<0.01	0.02	0.04	0.03	0.02	0.04	0.02
Residence size	0.01	0.01	0.04	0.02	0.01	0.04	0.01	0.01	0.02
Belief in the second wave	0.23	0.03	0.30^**^	0.22	0.03	0.29^**^	0.20	0.03	0.25^**^
Perceived personal risk				0.19	0.06	0.11^**^	0.18	0.06	0.10^**^
Perceived infectability				0.01	0.04	0.01	0.03	0.04	0.03
Germ aversion				0.24	0.03	0.26^**^	0.24	0.03	0.26^**^
Trust in science and scientists							0.21	0.04	0.17^**^
*R^2^*	0.13			0.20			0.23		
Δ*R^2^*	0.13^**^			0.08^**^			0.03^**^		

** *p* < 0.01.

Sociodemographics and belief in the second wave one accounted for 12.8% of the variation in intent to adhere to NPR. Women, older participants and those believing in the second wave had a higher intent to adhere to NPR. Adding perceived personal risk and perceived vulnerability to disease explained an additional 7.6% of criterion variance. This change was significant [*F* (3, 675) = 21.5; *p* < 0.001]. Participants who considered themselves or their significant others to be in high-risk COVID-19 group, and those with higher germ aversion had a higher adherence intent. Finally, trust in science and scientists entered in the third step explained additional 2.6% of intent to adhere to NPR. This change was also significant [*F* (1, 674) = 22.5; *p* < 0.001]. Gender, age, belief in the second wave, perceived personal risk, germ aversion, and trust in science and scientists were significant positive predictors in the final model with the belief in the second wave, germ aversion, and trust in science and scientists being the most important ones and accounting for 23% of the variance of the intent to adhere to NPR.

### Intent to Vaccinate

Again, a three-step multiple regression was conducted and the regression statistics are shown in Table 3. Sociodemographics and belief in the second wave accounted for 6.7% of the variation in the intent to vaccinate. Those believing in the second wave were more prone to vaccination. Adding perceived personal risk and perceived vulnerability to disease did not significantly contribute to the explanation of the criterion variance [*F* (3, 675) = 0.79; *p* = 0.50]. Finally, trust in science and scientists entered in the third step explained another 10% of the intent to vaccinate. This change was significant [*F* (1, 674) = 80.8; *p* < 0.001]. Only the belief in the second wave and trust in science and scientists were significant positive predictors in the final model, accounting for 16.9% of the variance in intent to vaccinate.

**Table 3:** Summary of hierarchical regression analysis for variables predicting the intent to vaccinate against COVID-19.

	Step 1			Step 2			Step 3		
Variable	*B*	*SE*	*β*	*B*	*SE*	*β*	*B*	*SE*	*β*
Gender	−0.18	0.11	−0.06	−0.20	0.11	−0.07	−0.01	0.11	−0.04
Age	<−0.01	0.01	−0.01	<−0.01	0.01	−0.02	<−0.01	0.01	−0.01
Education	0.05	0.08	0.03	0.06	0.08	0.04	0.03	0.07	0.02
Residence size	<0.01	0.03	0.01	0.01	0.03	0.01	−0.03	0.02	−0.04
Belief in second wave	0.33	0.05	0.25^**^	0.33	0.05	0.24^**^	0.24	0.05	0.18^**^
Perceived personal risk				0.11	0.12	0.04	0.09	0.11	0.03
Perceived infectability				−0.06	0.07	−0.04	−0.02	0.06	−0.01
Germ Aversion				0.06	0.06	0.04	0.07	0.06	0.05
Trust in Science and Scientists							0.70	0.08	0.33^**^
*R^2^*	0.07			0.07			0.17		
Δ*R^2^*	0.07^**^			<0.01			0.10^**^		

** *p* < 0.01.

## Discussion

This study aimed to explore the role of perceived personal risk and vulnerability to disease as threat appraisal variables and trust in science and scientists as a coping appraisal variable in intent to adhere to the official COVID-19 recommendations. Our model yielded a significant proportion of explained variance, and as expected – both types of appraisals contributed significantly to the explanation of variance of the intent to adhere to COVID-19 prevention measures.

### Adherence to NPRs vs. the Intent to Get Vaccinated

Women, older participants, and those believing in the second wave were more likely to adhere to the NPRs. Some studies have found age to be unrelated to voluntary compliance behavior, yet their participants were mostly young (mean age 27.2; Clark et al., 2020). A small positive correlation of age and intent to adhere is not entirely unexpected because the real risk of infection increases with age. BIS variables (perceived risk and vulnerability to disease) have explained 7.6% of the variance, while adding trust in science and scientists has explained additional 2.6%. Thus, gender, age, belief in the second wave, perceived risk, germ aversion, and trust in science and scientists are all significant positive predictors of the intent to adhere to NPR, with the belief in the second wave, germ aversion, and trust in science being the most important ones. The finding that germ aversion, but not perceived infectability predicted NPR adherence is in line with the recent finding of germ aversion being more associated with actions and perceived infectability with attentiveness (Makhanova and Shepherd, 2020). Our two sets of predictors represent two foundation blocks of the PMT, i.e., threat and coping appraisal, respectively. Protection motivation is considered synonymous with behavioral intent and is regarded as a strong mediator of the relation between both types of appraisal and subsequent behavior. The meta-analysis of 21 primary studies finds variables pertaining to each appraisal to be significantly correlated with the behavioral intention (Milne et al., 2000).

Regarding the intent to get vaccinated, only the belief in the second wave and trust in science proved to be significant predictors. Vaccination is a preventive measure whose mechanism of action is not easily understood by general public and which is held risky by lay-people. This is especially evident nowadays in the abundance of misinformation about vaccination, and during the mass vaccination campaign when each potential adverse effect is scrutinized and immediately picked by the social media platforms or mainstream news services. Even though the vaccine development is more transparent than ever, conspiracy theories thrive (Uscinski et al., 2020; Douglas, 2021). In order to suppress fears driven by the lack of understanding of the mechanisms underlying vaccines’ effects, one needs to have considerable trust in both, science/scientists and policy-makers. On the other hand, successful implementation of non-pharmacological measures does not require much knowledge and carries low risk. They are adhered to by those who are otherwise more sensitized to risks of contracting a disease, and show higher trait pathogen-avoidance. We believe this is the main reason why trust in science explains only an additional proportion of variance in adherence to the NPRs, and is, at the same time, the most significant predictor of adherence to the pharmacological measure.

The intent to vaccinate depends upon people’s perception of risk to contract a disease (Baumgaertner et al., 2020), thus we have expected for perceived vulnerability to predict intent to vaccinate as well. The reason why PVD did not predict the intent to vaccinate in this study might lie in the general nature of this measure: it measures pathogen avoidance as a trait, and this trait is usually expressed as disgust induced by (not necessarily conscious) detection of contamination risk. The intent to vaccinate is a deliberate, conscious choice, which might not be entirely reflected in one’s germ aversion or general infectability. A more specific measure regarding the perceived risks of contracting COVID-19 might prove to be more useful in explaining the willingness to vaccinate.

### Implications of This Study

This study was set as a synthesis of two different theoretical frameworks: the PMT and the BIS research. Both of them interpret certain motivations, and since BIS is more specifically oriented toward disease avoidance, it provided a more precise input for the conceptualization of the threat appraisal. Therefore, it comes as no surprise that BIS-informed threat appraisals have explained a significant share of the intent to adhere to NPR. Recent findings show that PVD: (1) has shifted significantly as a function of pandemic (Miłkowska et al., 2021; Stevenson et al., 2021), (2) can be manipulated experimentally (participants who read the coronavirus morbidity-mortality statistics and/or the government lifestyle regulations scored higher on PVD, compared to those who did not read such information; Bacon and Corr, 2020), and (3) predicts preventative behaviors (Makhanova and Shepherd, 2020; Shook et al., 2020; Stangier et al., 2021). It seems that tapping these evolved psychological mechanisms could prove instrumental for public interventions aiming to enhancing voluntary adherence to guidelines. For example, 55% of our participants reported perceiving themselves/their significant-others to be at high risk, and this perception correlated significantly with the intention to adhere to preventive measures. Thus interventions aiming at those not perceiving themselves to be at high risk might try to catalyze their personal threat appraisals by explaining the implications of large numbers: even though SARS-CoV-2 complications are more likely to occur among the identified high-risk populations, higher incidence of infection comes with a higher absolute numbers of fatalities even among young and healthy individuals.

Prophylactic behaviors seem to be common sense just as the findings of high-risk individuals to adhere more to the health guidelines. However, throughout the past year, a surprising amount of resistance to NPR was reported, often leading to societal polarization and culminating in street riots (Trian, 2020). Since humans are motivated to avoid disease, how did this resistance come into effect? Such motivations might be fueled by the appraisals stemming from the coping appraisals part of the PMT – here operationalized as the (mis)trust in science. Along with a pandemic, we are currently also dealing with an infodemic (World Health Organization, 2020b). The average reader is not well-equipped with skills and knowledge needed to differentiate between science and pseudoscience, false news, and checked facts. Interventions aimed at preventing a widespread gullibility will require meticulous planning and long-term goals. One such goal might be the augmentation of the public’s trust in science. In this study, the largest proportion of variance in the intent to vaccinate was explained by the trust in science. Mistrust in science, more specifically in vaccines, spreads across several domains: mistrust in benefits, worries of unforeseen effects, preference for natural immunity, and concerns about profiteering (Paul et al., 2020). Conspiratorial thinking and cognitive fallacies (including the so-called *argumentum ad big pharma*, see, e.g., Blaskiewicz, 2013) are deeply rooted in the popular narrative and it might prove counterproductive to address them directly. Elevating the trust in science as a general knowledge-augmenting process might induce less opposition. For example, one of the domains of mistrust in COVID-19 vaccine – “the preference for natural immunity” – might be changed when faced with the information that infecting a significant proportion of population might result in up to 30 million deaths worldwide (Randolph and Barreiro, 2020).

### Limitations of This Study

Apart from the well-established shortcomings of online surveys, such as respondents’ bias and unconscientious responses, this study features a potential flaw inherent to studies regarding behavioral intent – the so-called “intention-behavior gap” (Williams et al., 2015) or the discrepancy between intended and actual behavior. Future studies on preventive behavior and adherence to NPR should wisely incorporate measures of actual behavior as adherence criterion. It should be noted though, that a meta-analysis including approximately 30,000 participants showed that in general, increases in threat severity, vulnerability, response efficacy, and self-efficacy facilitates adaptive intentions or behaviors, irrespective of whether the measures were based on intentions or behaviors (Floyd et al., 2000), thus indicating the usefulness of PMT components for individual and community interventions. Furthermore, like any online sample, ours might have been biased regarding both demographic and relevant personality characteristics – women and participants higher on conscientiousness and agreeableness are more likely to participate (e.g., Bethlehem, 2010). However, our sample is rather diverse, with age spanning over 50 years, education ranging from elementary to PhD level, and a balanced gender proportion (44% men). Additionally, 87.7% of participants answered all of the questions.

In conclusion, we have found that both the BIS variables (entered as the threat appraisal within the model) and the trust in science (entered as the coping appraisal part of the model) contribute significantly to the intent to adhere to NPR, while only trust in science contributed significantly to the intent to vaccinate. Thus, interventions aimed at enhancing guidelines adherence should take into account that the psychological mechanisms underlying adherence to these two types of recommendations might not be identical, i.e., that the adherence to non-pharmacological measures is associated with threat and coping appraisal, while the intent to vaccinate is dominantly predicted by the response efficacy (an element of coping appraisal), such as trust in science or policy-makers.

## Data Availability Statement

The raw data supporting the conclusions of this article will be made available by the authors, without undue reservation.

## Ethics Statement

The studies involving human participants were reviewed and approved by Ethics Committee of the Dept. of Psychology, University of Zagreb. The patients/participants provided their written informed consent to participate in this study.

## Author Contributions

AV, IH, and MT contributed to the conception and design of the study and wrote the sections of the manuscript. MT organized the database. MT and IH performed the statistical analysis. AV and IH wrote the first draft of the manuscript. All authors contributed to the article and approved the submitted version.

### Conflict of Interest

The authors declare that the research was conducted in the absence of any commercial or financial relationships that could be construed as a potential conflict of interest.

## Footnotes

^1^The full scale is available at https://osf.io/j47qh/.
